# Effect of Countermovement Depth on the Neuromechanics of a Vertical Jump

**DOI:** 10.1155/2024/7113900

**Published:** 2024-06-06

**Authors:** Malachy P. McHugh, Josef Alexander Cohen, Karl F. Orishimo, Ian J. Kremenic

**Affiliations:** Nicholas Institute of Sports Medicine and Athletic Trauma, Lenox Hill Hospital, Northwell Health, MEETH a Division of Lenox Hill Hospital, 210 East 64 Street, New York, NY 10065, USA

## Abstract

The purpose of this study was to examine kinematic, kinetic, and muscle activation metrics during countermovement jumps (CMJs) with varying countermovement depths. The hypothesis was that a shallow countermovement depth would compromise jump height by disrupting neuromechanical control. Ten healthy men (age 26 ± 8 yr, height 1.81 ± 0.08 m, mass 83.5 ± 9.0 kg) performed maximal CMJs at self-selected countermovement depth (self-selected CMJ), at reduced countermovement depth (shallow CMJ), and at increased countermovement depth (deep CMJ). Three jumps were performed in each condition on force plates with ankle, knee, and hip motion recorded and electromyograms (EMG) recorded from the gluteus maximus (GM), vastus lateralis (VL), and medial gastrocnemius (MG) muscles. During CMJs, the knee flexion angle was recorded with an electrogoniometer. Jumpers were instructed to flex at least 15% less (shallow CMJ) and at least 15% more (deep CMJ) than the self-selected CMJs. Kinematic, kinetic, and EMG metrics were compared between the different CMJ depths using repeated measures ANOVA. Compared with self-selected CMJs, shallow CMJs had 26% less countermovement depth (*P* < 0.001, effect size 1.74) and the deep CMJs had 28% greater countermovement depth (*P* < 0.001, effect size 1.56). Jump height was 8% less for the shallow vs. self-selected CMJs (*P* = 0.007, effect size 1.09) but not different between self-selected and deep CMJs (*P* = 0.254). Shallow CMJs differed from self-selected CMJs at the initiation of the countermovement (unweighting). For self-selected CMJs, force dropped to 43% of body weight during unweighting but only to 58% for shallow CMJs (*P* = 0.015, effect size 0.95). During unweighting, VL EMG averaged 5.5% of MVC during self-selected CMJs versus 8.1% for shallow CMJs (*P* = 0.014, effect size 0.97). Percent decline in jump height with shallow versus self-selected CMJs was correlated with the difference in VL EMG during unweighting between shallow and self-selected CMJs (*r* = 0.651, *P* = 0.041). A deep countermovement prolonged the time to execute the jump by 38% (*P* < 0.010, effect size 1.04) but did not impair CMJ force metrics. In conclusion, self-selected countermovement depth represents a tradeoff between dropping the center of mass sufficiently far and executing the jump quickly. Unweighting at the initiation of a CMJ appears to be a critical element in the neuromechanics of the CMJ.

## 1. Introduction

A vertical jump is usually initiated by a countermovement whereby the center of mass is first lowered, then propelled vertically, hence the term countermovement jump (CMJ). The purpose of the countermovement is to store elastic energy for release during the propulsive phase of the jump [1]. Multiple studies have examined the effect of varying countermovement depths on a range of jump metrics. Decreasing the countermovement depth to less than the self-selected depth reduces jump height but increases the peak vertical force during the jump [2–8]. Increasing the countermovement depth to greater than the self-selected depth does not impair jump height [9] and, in fact, can improve jump height [3–5, 7, 8]. Surprisingly, none of these studies on the effect of countermovement depth examined the ground reaction force (GRF) profile of the countermovement. Furthermore, none of the studies examined the effect of countermovement depth on the kinetic and kinematic sequencing of the hips, knees, and ankles or the neuromuscular control of the sequencing.

The countermovement phase of a CMJ is composed of an unweighting phase and a braking phase, whereby an initial unloading of center of mass (unweighting) is followed by a rapid reloading to the low point of the countermovement (braking) [10]. None of the studies that manipulated the depth of the countermovement (2–9) examined the effects on any countermovement phase GRF metrics. While several studies reported a higher peak force when the depth of the countermovement was reduced, peak force typically occurs at the end of the braking phase or during the propulsive phase, not during the countermovement phase [10]. Examining the GRF force profile of the countermovement phase should provide more relevant information for understanding how countermovement depth affects CMJ biomechanics.

CMJ jump height is the result of the concerted actions of the muscles controlling hip extension, knee extension, and plantar flexion, in tandem with the storage and release of elastic energy from those muscle groups. However, the effect of countermovement depth on muscle activations, joint moments, and joint powers at the hip, knee, and ankle has not been examined. If a shallower countermovement results in an increase in peak GRF but a decrease in jump height, there is an impairment in the neuromechanics of the jump. Identifying where the impairments occur can provide important information for the optimal execution of CMJs and increase the understanding of the neuromechanics of CMJs.

Therefore, the purpose of this study was to compare the GRF profiles, muscle activation patterns, joint moments, and joint powers between shallow, self-selected, and deep CMJs. It was hypothesized that jump height would be decreased with a shallow countermovement, as has been shown previously, and that the shallow countermovement would impair the countermovement phase of the CMJ.

## 2. Methods

### 2.1. Participants

Ten healthy men (age 26 ± 8 yr, height 1.81 ± 0.08 m, mass 83.5 ± 9.0 kg) volunteered to participate. After explanation of procedures, all participants gave written informed consent and all procedures were approved by the Institutional Review Board (Northwell Health IRB# 19-0828). Participants were recreational or competitive athletes familiar with maximal vertical jump testing and were not currently injured nor had they had previous major surgery to a lower extremity.

### 2.2. Research Design and Procedures

This was a repeated measures single cohort study. Participants performed maximal CMJs at self-selected countermovement depth (self-selected CMJ), at reduced countermovement depth (shallow CMJ), and at increased countermovement depth (deep CMJ). Three jumps were performed in each condition on force plates with ankle, knee, and hip motion recorded and electromyograms (EMG) recorded from the gluteus maximus (GM), vastus lateralis (VL), and medial gastrocnemius (MG) muscles.

### 2.3. Countermovement Jumps

CMJs were performed with participants standing with each foot on a separate force plate (BTS Bioengineering, Quincy, MA). The GRF was recorded for all jumps at 1000 Hz. During CMJs, the hands were placed on the pelvis (akimbo) to eliminate arm swing. The self-selected depth CMJ was performed first with three trials performed. Subsequently, participants performed three shallow CMJs and then three deep CMJs. Jumpers were instructed to drop at least 15% less (shallow CMJ) and at least 15% more (deep CMJ) than the self-selected CMJs. The 15% threshold was chosen to ensure that the mechanics of the jump were sufficiently disrupted but not to an excess whereby the participants were unable to effectively jump. The knee flexion angle was recorded with an electrogoniometer (Penny & Giles, Gwent, United Kingdom) to provide feedback on CMJ depth so that participants could successfully surpass the target depth. Props were not used to provide a tactile cue when the participant reached the target depth as it might have altered the biomechanics of the jump. It took a couple of practice trials for participants to accurately achieve the countermovement depth for the shallow and deep CMJs. Once the participants were familiarized with the actual depth they had to achieve for shallow and deep countermovements, three trials were recorded. After each trial, the recorder confirmed that the countermovement depth was at least 15% greater than (deep) or less than (shallow) the self-selected depth. If these thresholds were not achieved, the trial was discarded and another trial was performed.

A total of 12 CMJ metrics were recorded from the GRF data for each jump. Each metric was averaged for the 3 trials at each countermovement depth. There were 7 metrics during the countermovement, 3 metrics during propulsion, and 2 performance metrics. The metrics during the countermovement were countermovement depth (m), countermovement duration (s), low force (% body weight), peak braking power (W), force at low position (% body weight), eccentric force (%BW), and eccentric stiffness (N.m^−1^). The metrics during the propulsive phase were peak propulsive force (% body weight), peak propulsive power (W), and propulsive phase duration (s). The performance metrics were jump height and reactive strength index (RSI). These metrics have been defined previously [10].

### 2.4. Motion Analysis

Kinematic data were recorded with a 10-camera motion capture system at 500 Hz with a 6 Hz low pass Butterworth filter (BTS Bioengineering, Quincy, MA). Retroreflective markers were placed on the lower extremities and trunk as follows: right and left acromia, right and left anterior superior iliac spine (ASIS), right and left greater trochanter, sacrum, right and left lateral femoral condyle, right and left medial femoral condyle, right and left lateral malleolus, right and left medial malleolus, and right and left base of the fifth metatarsal.

Net joint moments and powers were calculated for the ankle, knee, and hip by standard inverse dynamic techniques using specialized computer software (Visual 3D, C-Motion Inc, Rockville, Maryland, USA). All joint moments were reported as external moments and normalized to body mass. The peak moments and power for each joint and their timing relative to the low point of the countermovement were computed.

### 2.5. Electromyographic Measurements

During all CMJs, surface EMG signals from the gluteus maximus (GM), vastus lateralis (VL), and medial gastrocnemius (MG) were sampled at 1000 Hz from disposable Ag/AgCl passive dual electrodes (2.0 cm interelectrode distance) (Noraxon, Scottsdale, AZ, USA) using a 16-channel BTS FREEEMG 300 system (CMRR: 0.110 dB at 50–60 Hz; Input Impedance: 0.10 GV; BTS Bioengineering, Milan, Italy). For the GM, electrodes were placed in the center of the muscle belly between the lateral border of the sacrum and the greater trochanter. For the VL, electrodes were placed four fingerbreadths proximal to the superiolateral border of the patella along the assumed line of the fibers. For the MG, electrodes were placed longitudinally, one handbreadth below the popliteal crease on the medial mass of the muscle. The skin was shaved, cleaned, and lightly abraded prior to application of electrodes. Peak and average EMG signals, expressed as percentage of a maximum voluntary contraction (MVC), were computed for standing baseline (before initiation of the jump), initiation of unweighting to low force, low force to low position, and low position to take off. GM MVC was tested in prone with resisted isometric hip extension at 90° knee flexion. VL MVC was tested in sitting with resisted isometric knee extension at 80° knee flexion. MG MVC was tested in supine with resisted isometric plantar flexion at neutral plantar flexion and full extension at the knee.

### 2.6. Statistical Analyses

Kinematic, kinetic, and EMG metrics were compared between the different CMJ depths using repeated measures analysis of variance (ANOVA). For force plate metrics, countermovement depth was the only factor in the ANOVA. For moment, power, and EMG metrics, joint or muscle was added to the ANOVA with countermovement depth (3 × 3 repeated measures ANOVAs). Muscle (GM, VL, MG) by countermovement depth (shallow, self-selected, deep) ANOVAs were performed on average EMG activity for three separate jump phases: start of jump to low force, low force to low position, and low position to take off). ANOVA *P* values were adjusted for violations of sphericity using the Greenhouse–Geisser correction and are denoted by ^GG^. *F* ratios and partial eta squared (p eta^2^) values are reported with the *P* values for ANOVAs. For variables with a significant effect of countermovement depth, paired *t*-tests were used to identify significant differences between each of the three possible comparisons. Cohen's d effect sizes (ES) are reported for paired comparisons. Mean ± SD are reported in the tables and results section. Based on the reported variability for CMJ metrics (11) (coefficients of variation mostly <10%), it was estimated that with 10 participants, there would be 80% power to detect a difference in CMJ metrics of at least 10% at an alpha level of 0.05.

## 3. Results

### 3.1. CMJ Metrics

**Figure 1:**
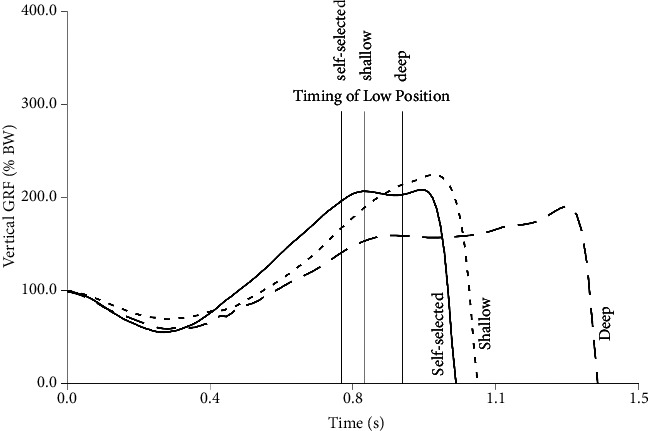
Ensemble averages of the GRF profiles of CMJs with a shallow, self-selected, or deep countermovement. The vertical lines indicate when low position (zero velocity) was reached.

The ensemble average GRF profiles for the CMJs with the difference countermovement depths are shown in Figure 1.

The effects of countermovement depth on all CMJ metrics are provided in Table 1. The shallow countermovement was 26% less than the self-selected countermovement, and the deep countermovement was 28% more than the self-selected. Jump height for the shallow countermovement was 7.5% less than self-selected (*P*=0.014) and 8.7% less than deep countermovement (*P*=0.007). Jump height was not different between self-selected and deep countermovement (*P*=0.363). By contrast, RSI was lower for the deep countermovement versus self-selected (*P*=0.004) and shallow (*P*=0.004) but not different between self-selected and shallow (*P*=0.352).

The only CMJ GRF metric that differed between the shallow and self-selected countermovement was low force, with less unweighting in the shallow countermovement (*P*=0.015). The deep countermovement resulted in longer countermovement (*P*=0.031) and propulsive durations (*P*=0.027) and decreased eccentric stiffness (*P*=0.013) compared with self-selected.

### 3.2. Joint Moments and Powers

The effect of countermovement depth on peak moments varied between joints (joint by depth *P*=0.025^GG^, *F* ratio 5.3, p eta^2^ 0.371). Peak ankle moment decreased with increasing countermovement depth (shallow 2.61 ± 0.51 Nm/kg, self-selected 2.39 ± 0.32 Nm/kg, deep 2.09 ± 0.25 Nm/kg, effect of depth *P*=0.003^GG^, *F* ratio 13.7, p eta^2^ 0.603). Countermovement depth did not affect peak hip (*P*=0.054, *F* ratio 3.5, p eta^2^ 0.277) and knee (*P*=0.066, *F* ratio 3.2, p eta^2^ 0.260) moments, but they tended to be higher with a deep countermovement (hip: deep 3.81 ± 1.14, self-selected 3.80 ± 0.86 Nm/kg Nm/kg, shallow 3.17 ± 0.70 Nm/kg; knee: deep 3.91 ± 0.82 Nm/kg, self-selected knee 3.44 ± 0.44 Nm/kg, shallow 3.69 ± 0.61 Nm/kg).

CMJ depth affected the timing of peak ankle moment (*P*=0.008, *F* ratio 6.4, p eta^2^ 0.414); peak ankle moment was delayed in the CMJs with a deep countermovement (323 ± 111 ms after low position) compared with the self-selected (182 ± 0.044 ms after low position, *P*=0.004, ES 1.22) and shallow (203 ± 164 ms after low position, *P*=0.010, ES 1.03).

Joint powers decreased with increasing countermovement depth (*P*=0.011, *F* ratio 5.8, p eta^2^ 0.394). This effect was not different between joints (Depth by Joint *P*=0.433) but was primarily apparent in the knee and ankle. Peak ankle powers during the CMJ with a shallow countermovement was higher than the CMJ with a deep countermovement (18.8 ± 3.2 vs. 15.5 ± 2.1 W/kg, *P*=0.008, ES 1.06). Hip and knee power were unaffected by countermovement depth (hip *P*=0.932: shallow 11.0 ± 3.9 W/kg, self-selected 11.2 ± 3.9 W/kg, deep 10.6 ± 3.4 W/kg; knee *P*=0.094: shallow 25.7 ± 4.7, self-selected 24.2 ± 4.5 W/kg, deep 23.6 ± 4.7 W/kg).

CMJ depth affected the timing of peak knee power (*P*=0.004, *F* ratio 7.8, p eta^2^ 0.465); peak knee power was delayed in the CMJs with a deep countermovement (366 ± 92 ms after low position) compared with self-selected (239 ± 31 ms after low position, *P*=0.004, ES 1.50) and shallow (247 ± 155 ms after low position, *P*=0.006, ES 1.12). Similarly, the timing of peak ankle power was delayed (*P*=0.006, *F* ratio 7.0, p eta^2^ 0.436) in the CMJs with a deep countermovement (378 ± 93 ms after low position) compared with self-selected (251 ± 30 ms after low position, *P*=0.001, ES 1.45) and shallow (259 ± 160 ms after low position, *P*=0.010, ES 1.03).

**Figure 2:**
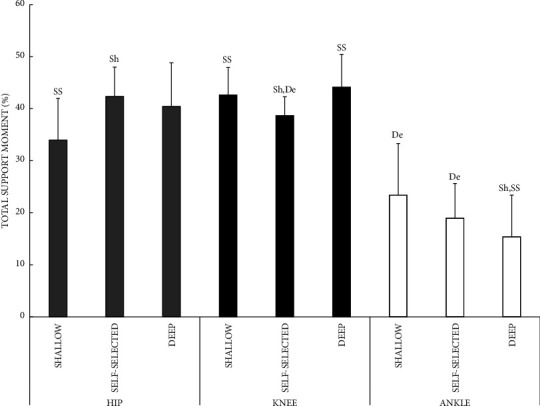
Hip, knee, and ankle contributions to peak total support moment during CMJs with a shallow, self-selected, or deep countermovement. Joint by countermovement depth *P*=0.021^GG^, *F* ratio 5.9, p eta^2^ 0.395; ^Sh^different from shallow, ^SS^different from self-selected, ^De^different from deep countermovement *P* < 0.05.

Countermovement depth affected the contributions of the hip, knee, and ankle to the peak total support moment (countermovement depth by joint *P*=0.001; Figure 2). The deep countermovement decreased the ankle contribution compared to the self-selected (*P*=0.035, ES 0.78) and shallow (*P*=0.012, ES 0.985) countermovement. The contribution of the knee to the peak total joint moment was lower for the self-selected countermovement depth compared with the deep (*P*=0.001, ES 1.45) and shallow (*P*=0.006, ES 1.13). The shallow countermovement decreased the contribution of the hip to the peak total joint moment compared with self-selected (*P*=0.013, ES 0.973).

The kinetic sequences between the hip, knee and the ankle were unaffected by countermovement depth. The peak hip and knee moments occurred almost simultaneously (self-selected: knee 27 ± 47 ms after hip, *P* = 0.097; shallow: knee 53 ± 100 ms after hip, *P* = 0.124; deep: knee 59 ± 344 ms after hip, *P* = 0.603). The peak ankle moment consistently occurred after peak knee moment (self-selected 135 ± 55 ms after knee, *P* < 0.001; shallow 80 ± 61 ms after knee, *P* = 0.003; deep 229 ± 97 ms after knee, *P* < 0.001).

### 3.3. Muscle Activations

The effect of countermovement depth on muscle activation (Table 2) varied between the 3 muscles during the time from initiation of unweighting (start of jump) to maximum unweighting (low force) (depth by muscle *P* = 0.020^GG^) and during the propulsive phase of the jump (low position to take off) (depth by muscle *P* = 0.017). The effect of depth on muscle activations during the time from low force to low position was not different between muscles (*P* = 0.301).

#### 3.3.1. CMJ Initiation to Low Force

**Figure 3:**
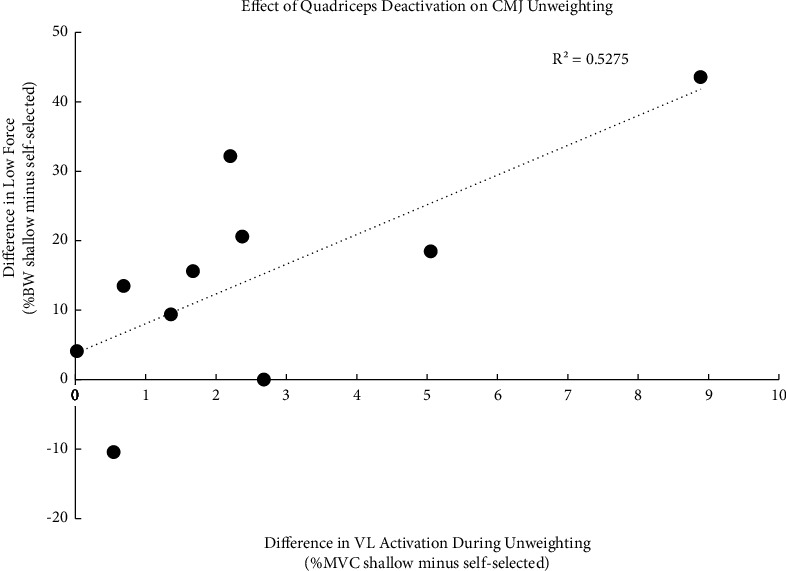
Correlation between the differences in unweighting for shallow versus self-selected countermovements (*y* axis) and the differences in VL activation during unweighting for shallow and self-selected countermovements (*x* axis). *Y* axis is low force for shallow minus low force for self-selected; a positive number means less unweighting in the shallow versus self-selected countermovement. *X* axis is the difference in average VL EMG amplitude (%MVC) from initiation of unweighting to low force for shallow minus self-selected; a higher number means greater activation during the initiation of the shallow countermovement. VL deactivation during the countermovement explains 52.75% (*R*^2^) of the difference in unweighting (low force) between shallow and self-selected countermovements (*P*=0.017).

From the initiation of unweighting to low force, the effect of countermovement depth was specific to the VL (muscle by countermovement depth *P* = 0.002). Average VL EMG during this period was lowest for the self-selected countermovement (5.5 ± 4.3%) and highest for the shallow countermovement (8.1 ± 5.1%; effect of countermovement depth *P* = 0.023, self-selected vs. shallow *P* = 0.014). Across all three countermovement depths, VL activity from the initiation of unweighting to low force (6.8 ± 4.7%) was higher than GM (2.4 ± 0.9%) and MG (1.6 ± 0.6%) activity (effect of muscle *P* = 0.001, VL vs. GM *P* = 0.017, VL vs. MG *P* = 0.006). The difference in VL activation between shallow and self-selected countermovements was strongly correlated with the difference in low force between shallow and self-selected countermovements (*R*^2^ = 0.5275, *P* = 0.017, Figure 3); quadriceps deactivation during the initiation of the countermovement explained the greater unweighting in the self-selected versus shallow countermovement. Furthermore, the difference in VL EMG during unweighting between shallow and self-selected CMJs was correlated with percent decline in jump height with shallow versus self-selected CMJs (*r* = 0.651, *P* = 0.041).

#### 3.3.2. From Low Force to Low Position

From low force to low position average EMG activity was also higher for VL (41 ± 21%) versus GM (6.3 ± 3.2%, *P* < 0.001) and MG (10.9 ± 10.4%; effect of muscle *P* < 0.001). Across all three muscles, EMG activity from low force to low position was lower for the deep countermovement (14.7 ± 7.3%) versus self-selected (20.1 ± 7.6%, *P* < 0.001) and shallow (23.4 ± 16.1%, *P*=0.019; effect of countermovement depth *P*=0.015).

#### 3.3.3. From Low Position to Take Off (Propulsive Phase)

From low position to take off, average EMG activity was also higher for VL (135.1 ± 49.3%) versus GM (41.0 ± 10.4%, *P* < 0.001) and MG (75.6 ± 22.1%, *P*=0.001; effect of muscle *P* < 0.001). EMG activity from low position to take off was affected by countermovement depth (*P*=0.007), but this effect differed between muscles (countermovement depth by muscle *P*=0.017). GM activity was lowest for the deep countermovement (36.5 ± 10.2%) and highest for the shallow countermovement (44.6 ± 13.0%, effect of countermovement depth *P*=0.014, deep vs. shallow *P*=0.009). However, countermovement depth did not affect VL EMG activity from low position to toe off (deep 130.3 ± 46.1%, self-selected 136.0 ± 54.7%, shallow 139.1 ± 52.6%, *P*=0.506). The effect of countermovement depth on EMG activity from low position to take off was most apparent in the MG (*P* < 0.001). MG activity for the deep countermovement (60.1 ± 21.9%) was lower than self-selected (78.9 ± 25.7%, *P*=0.009) and shallow (87.9 ± 24.4%, *P* < 0.001).

## 4. Discussion

This is the first study to comprehensively examine the role of countermovement depth on CMJ force plate metrics in conjunction with multijoint moments and power and lower extremity muscle activation patterns. By manipulating just one aspect of the CMJ (countermovement depth), the results provide a clearer understanding of the neuromechanics of CMJs.

### 4.1. CMJ Metrics

The two performance metrics (jump height, modified RSI) were affected differently by altering countermovement distance. A shallow countermovement compromised jump height but a deep countermovement did not. This is consistent with several previous studies [2–8]. A deep countermovement compromised RSI but a shallow countermovement did not. Similarly, Pérez-Castilla et al. 2023 [6] found that RSI was compromised by a deep countermovement but jump height was not. Thus, the self-selected countermovement depth was optimal in terms of the height jumped and the time required to execute the jump. From a performance perspective, being able to jump high quickly would be preferred and identifies an efficient execution of the task.

The deep countermovement primarily compromised the time taken to execute the jump (36% longer countermovement duration, 42% longer propulsive duration compared to self-selected). The shallow countermovement primarily compromised unweighting at the initiation of the jump. There was less unweighting in the shallow versus self-selected countermovement (low force: shallow 58 ± 26% of body weight, self-selected 43 ± 24% of body weight). This highlights the importance of unweighting at the initiation of a CMJ. Surprisingly, the magnitude of unweighting is rarely reported even though unweighting is the first action in a CMJ.

### 4.2. Joint Moments and Powers

In terms of the contribution of each joint to peak total support moment, a shallow countermovement decreased the contribution from the hip and increased the contribution from the knee when compared with self-selected depth. The opposite pattern was apparent during the deep countermovement, with a decreased contribution from the ankle and an increased contribution from the knee.

A lower peak power at the knee and ankle with increasing countermovement depth was primarily a timing issue with longer time to reach peak knee and ankle moments and power with increasing countermovement depth.

### 4.3. Muscle Activation

Overall, CMJs were a quadriceps dominant activity, with the highest activations seen in the propulsive phase of the jump. This quadriceps dominance in the propulsive phase of a CMJ is consistent with previous findings [11, 12]. However, it was at the initiation of the jump, during unweighting, where the effect of quadriceps activation on CMJ performance was most apparent. VL activation during unweighting was lower during the self-selected versus shallow countermovement (5.5% vs. 8.1% MVC). This deactivation in the self-selected versus shallow countermovement was strongly correlated with the greater unweighting seen with the self-selected versus shallow countermovement (Figure 3). Thus, the ability to deactivate antigravity muscles at the initiation of a CMJ appears to dictate the subsequent jump performance.

The effect of countermovement depth on MG activation paralleled the effects on total support moment, i.e., lower contribution to total support moment from the ankle during the deep countermovement. MG activation during the propulsive phase was lower for the deep countermovement (60% MVC) compared with the self-selected (79%) and shallow (88%) countermovement. However, the effect of countermovement depth on GM activity did not parallel the effects on total support moment. Compared to the self-selected, a shallow countermovement decreased the contribution of the hip to total support moment. By contrast, GM activity was not different between self-selected and shallow countermovements. This indicates some neuromechanical inefficiency at the hip during the shallow countermovement.

### 4.4. Conclusion

A shallow countermovement decreased jump height but did not affect the force and power produced during the propulsive phase. Jump height with a shallow countermovement was impaired by ineffective unweighting at the initiation of the jump. Unweighting appears to be a critical element in the neuromechanics of the CMJ. By contrast, a deep countermovement did not impair jump height but prolonged the time to execute the jump (38% longer). Therefore, the self-selected countermovement depth represents the best option from a practical perspective as it is a tradeoff between dropping the center of mass sufficiently far and executing the jump quickly.

## Figures and Tables

**Table 1:** Differences in jump metrics between shallow, self-selected, and deep countermovements (mean ± SD, ES = effect size).

	CMJ group			ANOVA *P* value *F ratio/p* *eta*^2^
	Shallow	Self-selected	Deep	
*Countermovement metrics*				
Countermovement depth (m)	0.269 ± 0.056^SS,De^ *ES* 1.74^*SS*^	0.364 ± 046^Sh,De^ *ES* 1.56^*De*^	0.467 ± 0.059^Sh,SS^ *ES* 3.26^*SS*^	<0.001 *53.3/0.856*
Countermovement duration (s)	0.765 ± 0.321^De^ *ES* 0.73^*Sh*^	0.701 ± 194^De^ *ES* 0.81^*Sh*^	0.950 ± 0.370^Sh,SS^	0.032 *4.2/0.318*
Low force (%BW)	58 ± 26%^SS^	43 ± 24%^Sh^ *ES* 0.95^*Sh*^	51 ± 28%	0.008 *6.3/0.412*
Peak braking power (W)	840 ± 444	1104 ± 435	1059 ± 681	0.106 *2.6/0.221*
Force at low position (%BW)	207 ± 45%	212 ± 27%	199 ± 59%	0.609 0.5/0.054
Eccentric force (%BW)	149 ± 66%	169 ± 48%	152 ± 80%	0.338 *1.2/0.113*
Eccentric stiffness (N^.^m^−1^)	4822 ± 2475^De^ *ES* 1.29^*De*^	3876 ± 1221^De^ *ES* 0.97^*De*^	2660 ± 1263^Sh,SS^	0.001 *9.7/0.519*

*Propulsive metrics*				
Peak propulsive force (%BW)	246 ± 43%	224 ± 20%	225 ± 36%	0.096 *2.7/0.229*
Peak propulsive power (W)	4320 ± 690	4155 ± 887	4224 ± 774	0.293 *1.3/0.128*
Propulsive phase duration (s)	0.269 ± 0.059^De^ *ES* 2.35^*De*^	0.301 ± 0.029^De^ *ES 1.49*	0.428 ± 0.092^Sh,SS^	<0.001 *30.6/0.773*

*Performance metrics*				
Jump height (m)	0.345 ± 0.048^SS,De^	0.373 ± 0.044^Sh^ *ES* 1.09^*Sh*^	0.378 ± 0.056^Sh^ *ES* 0.78^*Sh*^	0.008^GG^ *8.9/0.498*
RSI (arbitrary units)	0.362 ± 0.109^De^ *ES 1.21*	0.386 ± 0.084^De^ *ES 1.21*	0.300 ± 0.107^Sh,SS^	0.003 *8.5/0.485*

BW = body weight; RSI = reactive strength index (jump height/time from jump initiation to take off); ^Sh^significantly different from Shallow group, ^SS^significantly different from the self-selected group, ^De^significantly different from the deep group (*P* < 0.05). ^GG^ANOVA *P* value corrected for sphericity violation using a Greenhouse–Geisser correction. The partial eta squared (*p* eta^2^) values for the ANOVAs are reported with the F ratio below the ANOVA *P* value (F ratio/p eta^2^). Cohen's d effect sizes (ES) are reported for significant pairwise comparisons.

**Table 2:** Average EMG amplitude (%MVC) for GM, VL, and MG for the shallow, self-selected, and deep countermovements from the start of unweighting to low force, from low force to low position, and from low position to take-off.

		Start to low force	Low force to low position	Low position to take-off *P*=0.288
GM	Shallow	1.7 ± 0.6%	8.1 ± 4.7%^De^ *ES 1.10*	44.6 ± 13.0%^De^ *ES 1.05*
	Self-selected	1.5 ± 0.5%	7.2 ± 5.0%^De^ *ES 0.821*	42.0 ± 10.7%
	Deep	1.6 ± 0.8%	3.6 ± 1.2%^Sh,SS^	36.5 ± 10.2%^Sh^
Effect of countermovement depth *(F ratio/p eta2)*	*P*=0.288 *1.3/0.129*	*P*=0.010 *6.0/0.400*	*P*=0.014 *5.5/0.380*	

VL	Shallow	8.1 ± 5.1%^SS^ *ES 0.97*	46.7 ± 28.4%^De^ *ES 1.06*	139.1 ± 52.6%
	Self-selected	5.5 ± 4.3%^Sh^	42.7 ± 18.4%^De^ *ES 1.26*	136.0 ± 54.7%
	Deep	6.8 ± 5.9%	33.7 ± 19.0%^Sh,SS^	130.3 ± 46.1%
Effect of countermovement depth *(F ratio/p eta2)*	*P*=0.023 *4.7/0.343*	*P*=0.008 *6.4/0.416*	*P*=0.506 *0.7/0.073*	

MG	Shallow	2.2 ± 0.9%	15.3 ± 21.4%	87.9 ± 24.4%^De^ *ES 1.81*
	Self-selected	2.8 ± 1.5%	10.5 ± 8.5%	78.9 ± 25.7%^De^ *ES 1.05*
	Deep	2.4 ± 0.9%	6.9 ± 4.2%	60.1 ± 21.9%^Sh,SS^
Effect of countermovement depth *(F ratio/p eta2)*	*P*=0.154 *2.1/0.187*	*P*=0.227 *1.6/0.152*	*P* < 0.001 *15.0/0.625*	

Depth by muscle *(F ratio/p eta2)*	*P*=0.020^*GG*^ *5.1/0.360*	*P*=0.301 *1.3/0.123*	*P*=0.017 *3.5/0.279*	

^Sh^Different from shallow; ^SS^different from self-selected; ^De^different from deep, *P* < 0.05. ^GG^ANOVA *P* value corrected for sphericity violation using a Greenhouse–Geisser correction. The partial eta squared (*p* eta2) values for the ANOVAs are reported with the *F* ratio below the *P* ANOVA value (*F* ratio/*p* eta2). Cohen's d effect sizes (ES) are reported for significant pairwise comparisons.

## Data Availability

The data will be made available upon request.
